# MRI-Derived Evaluation of Liver Function and Its Extension to K-Edge Photon-Counting CT: Focus on Gadolinium-Based Functional Biliary Imaging

**DOI:** 10.3390/diagnostics16172740

**Published:** 2026-08-26

**Authors:** Luigi Asmundo, Ilaria Vicentin, Gaia Ghilardi, Caterina Beatrice Monti, Andrea Vanzulli, Francesco Rizzetto, Leonardo Mariani, Simone Steffani, Leonardo Centonze, Chiara Mazzarelli, Domenico Albano, Stefano Di Sandro, Angelo Vanzulli

**Affiliations:** 1 Department of Radiology, ASST Grande Ospedale Metropolitano Niguarda, Piazza Ospedale Maggiore 3, 20162 Milan, Italy; ilaria.vicentin@ospedaleniguarda.it (I.V.); gaia.ghilardi@ospedaleniguarda.it (G.G.); caterinabeatrice.monti@ospedaleniguarda.it (C.B.M.); andrea.vanzulli@ospedaleniguarda.it (A.V.); leonardo.mariani@ospedaleniguarda.it (L.M.); domenico.albano@ospedaleniguarda.it (D.A.); angelo.vanzulli@ospedaleniguarda.it (A.V.); 2 Diagnostic Imaging Department, University of Rome Tor Vergata, 00133 Rome, Italy; simone.steffani@ptvonline.it; 3 Department of General Surgery and Transplantation, ASST Grande Ospedale Metropolitano Niguarda, Piazza Ospedale Maggiore 3, 20162 Milan, Italy; leonardo.centonze@ospedaleniguarda.it (L.C.); stefano.disandro@ospedaleniguarda.it (S.D.S.); 4 Gastroenterology and Hepatology Department, Liver Unit, ASST Grande Ospedale Metropolitano Niguarda, Piazza Ospedale Maggiore 3, 20162 Milan, Italy; chiara.mazzarelli@ospedaleniguarda.it; 5 Dipartimento di Scienze Biomediche, Chirurgiche ed Odontoiatriche, Università degli Studi di Milano, Via della Commenda 10, 20122 Milan, Italy; 6 Department of Oncology and Hemato-Oncology, Università degli Studi di Milano, Via Festa del Perdono 7, 20122 Milan, Italy

**Keywords:** gadoxetic acid, hepatobiliary MRI, liver function, functional biliary imaging, photon-counting CT, K-edge imaging, quantitative MRI, liver fibrosis

## Abstract

Accurate assessment of liver function is essential in the management of chronic liver disease, hepatobiliary malignancies, and surgical planning. In recent years, hepatobiliary magnetic resonance imaging (MRI) using gadolinium-based contrast agents has evolved from a purely morphological technique into a quantitative functional imaging modality capable of evaluating hepatocyte uptake, biliary excretion, and regional liver function. Quantitative MRI-derived biomarkers, including relative liver enhancement, liver-to-spleen ratio, T1 relaxometry, and pharmacokinetic modeling, have demonstrated significant correlations with established liver function tests and postoperative outcomes. At the same time, the introduction of photon-counting computed tomography (PCCT) and K-edge imaging has opened new perspectives in quantitative imaging. Energy-resolved photon-counting technology has demonstrated gadolinium discrimination and material decomposition under selected experimental conditions, raising the hypothesis that aspects of hepatobiliary contrast distribution might eventually be investigated with computed tomography (CT). However, reliable K-edge detection and quantitative hepatobiliary gadolinium imaging after standard clinical administration have not yet been established in humans. Phantom, simulation, preclinical, and limited non-hepatobiliary human studies have investigated gadolinium K-edge imaging, but direct evidence for functional hepatobiliary PCCT in humans remains lacking. This review aims to provide a comprehensive overview of MRI-derived evaluation of liver function, with particular attention to the experimental evidence exploring whether selected MRI-derived concepts might ultimately be investigated using PCCT and K-edge imaging, highlighting current evidence, technical challenges, translational applications, and future perspectives in precision hepatobiliary diagnostics.

## 1. Introduction

The accurate assessment of liver function represents a cornerstone in the management of a wide spectrum of hepatic diseases, ranging from chronic liver disease and cirrhosis to primary and secondary malignancies. Traditional liver function tests, including serum biomarkers such as bilirubin, albumin, and transaminases, as well as dynamic tests like indocyanine green (ICG) clearance, provide only global information and lack spatial resolution [1,2,3]. This limitation is particularly relevant in clinical scenarios where liver function is heterogeneous, such as in cirrhosis, in chemotherapy-associated liver injury, or following portal vein embolization, where regional functional differences may critically influence treatment decisions [2].

Over the past two decades, imaging has undergone a paradigm shift, evolving from a purely morphological discipline to one capable of providing functional and quantitative information. Among available modalities, magnetic resonance imaging (MRI) using hepatobiliary contrast agents has emerged as a powerful tool for the non-invasive evaluation of liver function [4,5]. Hepatobiliary contrast agents such as gadoxetate disodium (Gd-EOB-DTPA) and gadobenate dimeglumine (Gd-BOPTA) allow for the visualization of hepatocyte uptake and biliary excretion, thereby enabling the assessment of hepatocellular function at both global and regional levels [6]. Numerous studies have demonstrated strong correlations between MRI-derived parameters and established liver function tests, supporting the clinical relevance of this approach [7,8,9,10,11].

In parallel, technological advances in computed tomography (CT), particularly the development of photon-counting CT (PCCT), have opened new avenues for functional imaging. Unlike conventional energy-integrating CT systems, photon-counting detectors can measure the energy of individual photons, enabling spectral imaging and material decomposition. This capability provides the physical basis for K-edge imaging, whereby elements such as gadolinium may be discriminated under appropriate detector, concentration, and acquisition conditions [12,13].

The convergence of these developments raises an intriguing possibility: the extension of MRI-derived functional liver imaging concepts to CT through K-edge photon-counting technology [14,15,16]. These developments raise the hypothesis that gadolinium distribution might eventually be assessed using photon-counting CT. However, physical gadolinium detection, K-edge discrimination, quantitative material decomposition, and functional hepatobiliary imaging represent distinct technical and biological endpoints. Whether gadolinium concentrations achieved in the liver and biliary system after clinically approved hepatobiliary contrast administration are sufficient for reliable K-edge detection and quantitative imaging in humans remains uncertain.

This review provides a comprehensive overview of MRI-derived liver function assessment, with a focus on gadolinium concentration in the biliary system and functional biliary imaging. It further explores the emerging role of PCCT and K-edge imaging, discussing their potential applications, limitations, and future directions in the field of medical diagnostics.

## 2. Hepatobiliary Contrast Agents and Their Pharmacokinetics

Hepatobiliary contrast agents represent a unique class of gadolinium-based contrast agents characterized by their dual behavior, combining extracellular distribution with hepatocyte-specific uptake and biliary excretion [17]. Gd-EOB-DTPA, in particular, has been extensively studied due to its favorable pharmacokinetic profile, with approximately 50% of the administered dose being taken up by hepatocytes and excreted into the bile, while the remainder is eliminated via the kidneys [18]. Gd-BOPTA exhibits a lower degree of hepatobiliary excretion, typically ranging between 3% and 5%, but still provides delayed hepatobiliary enhancement [19].

The uptake of gadoxetate is mediated by organic anion transporting polypeptide 1B1 (OATP1B1) and OATP1B3, located on the sinusoidal membrane of hepatocytes. Once inside the cell, the contrast agent is transported into bile canaliculi via adenosine triphosphate (ATP)-dependent transporters such as multidrug resistance-associated protein 2 (MRP2). This process is influenced by several factors, including hepatocyte integrity, transporter expression, sinusoidal perfusion, intracellular metabolism, and bile flow dynamics [20]. Alterations in these mechanisms are commonly observed in liver disease [20].

**Figure 1:**
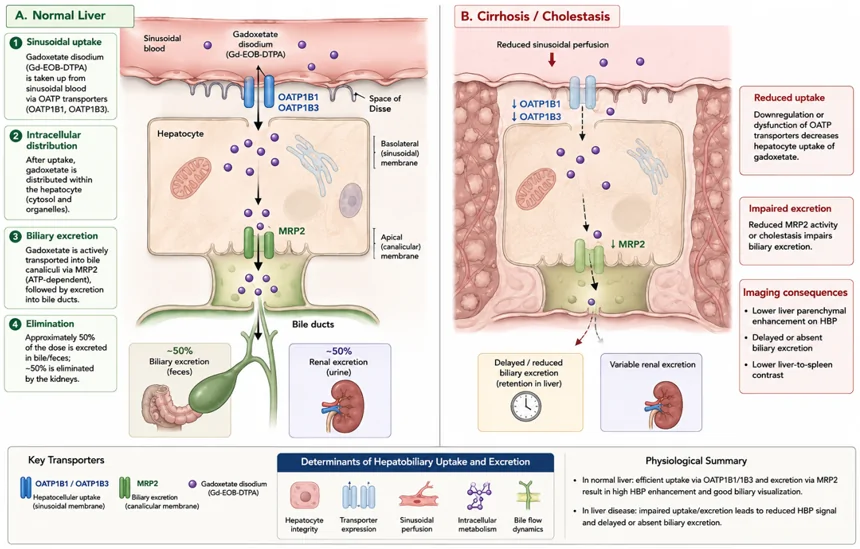
Physiological mechanisms of hepatobiliary gadolinium uptake and biliary excretion. Schematic illustration of hepatocyte uptake mediated by OATP1B1/OATP1B3 transporters, intracellular distribution, and ATP-dependent biliary excretion through MRP2. The figure also summarizes the principal mechanisms responsible for reduced hepatobiliary enhancement in chronic liver disease.

For instance, downregulation of OATP transporters in cirrhosis leads to reduced hepatocyte uptake, while impaired MRP2 function can result in delayed biliary excretion. These pathophysiological changes are directly reflected in hepatobiliary phase imaging, providing a non-invasive window into liver function [21,22]. The physiological and molecular mechanisms underlying hepatobiliary gadolinium uptake and biliary excretion are summarized in Figure 1.

The hepatobiliary phase is typically acquired 15–20 min after contrast injection for Gd-EOB-DTPA. During this phase, normal liver parenchyma appears hyperintense due to intracellular accumulation of gadolinium, whereas lesions lacking functional hepatocytes remain hypointense. This contrast behavior has been extensively exploited for lesion detection and characterization, particularly in hepatocellular carcinoma and liver metastases [23].

**Figure 2:**
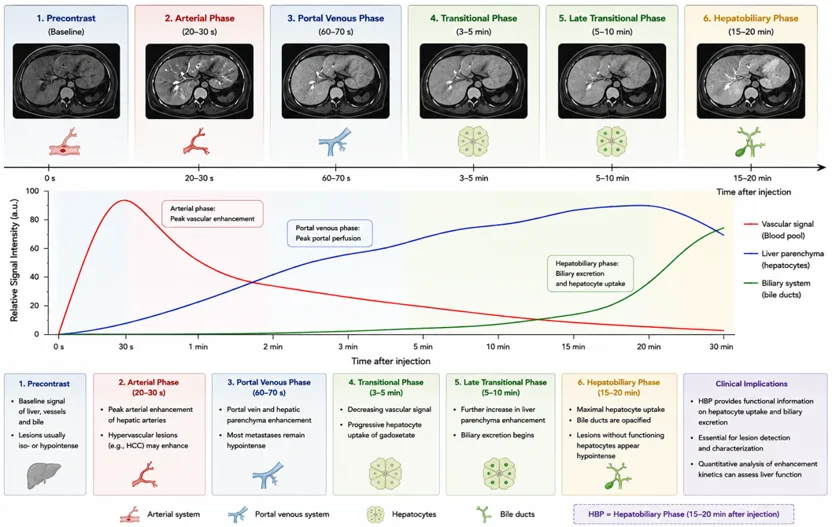
Temporal evolution of hepatobiliary contrast enhancement following Gd-EOB-DTPA administration. Schematic representation of the arterial, portal venous, transitional, and hepatobiliary phases, illustrating the dynamic changes in gadolinium distribution within the vascular, hepatic parenchymal, and biliary compartments.

Beyond lesion characterization, the dynamic evolution of hepatobiliary enhancement provides critical information regarding hepatocyte uptake and biliary excretion kinetics [20]. The temporal changes in signal intensity from the vascular, parenchymal, and biliary compartments throughout the different phases of imaging are illustrated in Figure 2.

The clinical success of hepatobiliary contrast agents has also stimulated interest in molecular imaging and theranostic applications. Because hepatocyte uptake depends on transporter activity, contrast enhancement can indirectly reflect the expression of transport proteins. This has implications not only for liver function assessment but also for tumor biology. For example, the loss of OATP expression in hepatocellular carcinoma contributes to the typical hypointensity observed on hepatobiliary phase imaging and may correlate with molecular subtype and tumor aggressiveness [24,25,26].

Another important consideration concerns the timing of hepatobiliary phase acquisition. Although 20 min is generally considered standard for Gd-EOB-DTPA, delayed imaging up to 60–120 min may improve visualization in patients with impaired liver function or cholestatic disease [27]. Delayed hepatobiliary imaging can increase sensitivity for bile leak detection and may enhance assessment of biliary excretion kinetics [28].

## 3. Quantitative MRI for Liver Function Assessment

Quantitative MRI approaches aim to move beyond qualitative assessment by providing objective metrics of liver function. Among the most commonly used parameters are relative liver enhancement (RLE), liver-to-spleen contrast ratio, hepatic uptake index (HUI), contrast enhancement index, and T1 relaxometry-based measurements [29]. These hepatobiliary phase-derived metrics have demonstrated excellent reproducibility, particularly for RLE with an inter-observer intraclass correlation coefficient (ICC) up to 0.979, and showed significant correlations with established liver function scores, including the albumin–bilirubin (ALBI) score, the Model for End-Stage Liver Disease (MELD), and Child–Pugh (all *p* < 0.001), with good performance in identifying patients with impaired liver function; in particular, for MELD ≥ 15, the area under the curve (AUC) was up to 0.782 [29].

RLE is typically calculated as the percentage increase in liver signal intensity between precontrast and hepatobiliary phase imaging [30]. More recently, Kim et al. [31] demonstrated that MRI-derived quantitative biomarkers, particularly the relative HUI and its body weight-corrected version, were independent predictors of posthepatectomy liver failure and showed higher predictive performance than RLE, ICG plasma disappearance rate, and future remnant liver volume in that cohort. Similarly, the liver-to-spleen ratio has been associated with Child–Pugh and MELD scores, providing a non-invasive surrogate for liver function [32,33].

The HUI incorporates vascular input information and normalizes hepatic enhancement relative to blood pool enhancement [7]. In recent years, T1 mapping techniques have gained increasing attention because they allow quantitative measurement of T1 relaxation times before and after contrast administration. Reduction in T1 relaxation time after Gd-EOB-DTPA administration is a reproducible biomarker of hepatocyte uptake across different magnetic field strengths and accurately differentiates cirrhotic from non-cirrhotic livers. More advanced T1 mapping-derived parameters, including the hepatic extraction fraction and hepatic uptake rate constant, show excellent correlation with ICG-R15 and may provide a more accurate quantitative assessment of liver function than conventional signal intensity-based methods [34,35].

More advanced techniques involve dynamic contrast-enhanced MRI and pharmacokinetic modeling. These approaches enable the estimation of parameters such as the hepatic uptake rate constant, biliary excretion rate, extracellular volume fraction, and intracellular contrast concentration [36,37]. Such models provide a more comprehensive characterization of liver function, capturing both uptake and excretion processes.

No single MRI-derived biomarker has demonstrated universal superiority across clinical applications. Signal-intensity-based parameters such as RLE are simple and reproducible within standardized acquisitions but remain dependent on scanner platform, sequence parameters, coil sensitivity, and acquisition timing. Liver-to-spleen ratios and CEI introduce internal normalization, potentially reducing some technical variability, although measurements may be affected by the characteristics of the selected reference tissue and ROI methodology. HUI incorporates blood-pool normalization and has shown promising discrimination in surgical cohorts but remains dependent on acquisition timing and ROI definition. T1-based measurements provide a more intrinsically quantitative assessment of hepatocyte-related contrast uptake, although values remain influenced by field strength, sequence implementation, and vendor-specific methodology. Pharmacokinetic modeling can separate uptake and excretion components and provides greater physiological specificity, but requires more complex dynamic acquisitions and post-processing and currently remains predominantly a research technique. Biomarker selection should therefore depend on the clinical question, available acquisition methodology, and level of external validation rather than assuming interchangeability or universal superiority.

Importantly, MRI allows regional analysis, enabling the assessment of segmental liver function. This is particularly relevant in surgical planning, where the functional capacity of the future liver remnant is a critical determinant of postoperative outcome. Several studies have reported greater discrimination with selected MRI-derived functional biomarkers than with volumetric assessment in specific cohorts. For example, in one multicenter cohort, HUI showed an AUC of 0.758 compared with 0.628 for standardized future liver remnant volume, while a model combining HUI with MELD 3.0 showed an AUC of 0.803 [7]. Similarly, the remnant liver contrast enhancement ratio, a study-specific signal-intensity-based metric distinct from RLE, showed an AUC of 0.78 for prediction of posthepatectomy liver failure [7,38]. Differences in AUC between imaging biomarkers and conventional clinical or volumetric models should be interpreted as differences in discrimination within the populations studied and do not, in isolation, establish statistical superiority, incremental clinical utility, or improved patient outcomes. Formal model comparison, assessment of calibration and reclassification, external validation, and ideally evaluation of clinical decision impact are required before an imaging biomarker can be considered to provide incremental value over established approaches.

**Figure 3:**
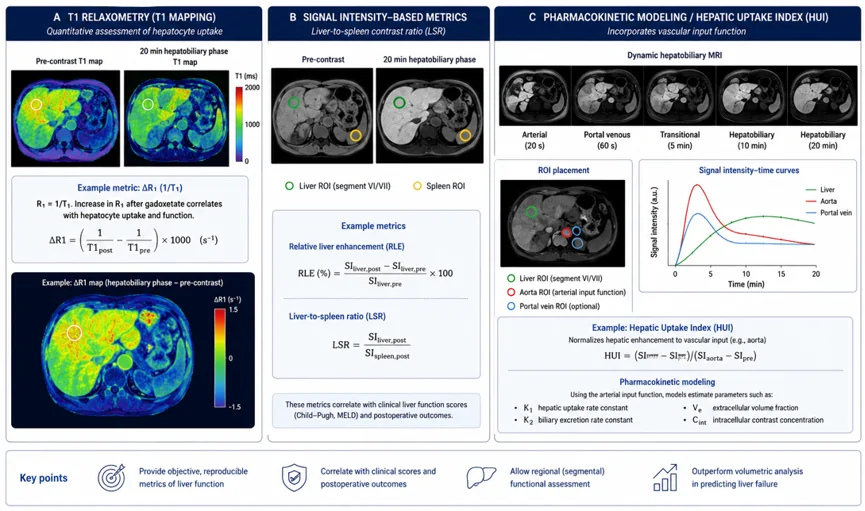
Quantitative MRI biomarkers for liver function assessment. Overview of the principal quantitative MRI-derived biomarkers, including relative liver enhancement (RLE), liver-to-spleen ratio, hepatic uptake index (HUI), T1 mapping, and pharmacokinetic modeling. Representative parametric maps and schematic illustrations of uptake and excretion kinetics are shown.

The principal quantitative biomarkers currently used for MRI-derived liver function assessment, together with schematic examples of parametric mapping and pharmacokinetic modeling, are illustrated in Figure 3.

The emergence of artificial intelligence and radiomics has further expanded the potential of quantitative hepatobiliary MRI. Machine learning models integrating radiomic features, quantitative MRI biomarkers, enhancement kinetics, and clinical variables have demonstrated high diagnostic performance for the non-invasive assessment of liver fibrosis, disease severity, and postoperative liver function [39,40]. In particular, MRI-based radiomics has achieved diagnostic performance comparable to magnetic resonance elastography for fibrosis staging, while recent MRI-based machine learning approaches have shown excellent accuracy in stratifying metabolic dysfunction-associated steatotic liver disease [39,40]. Deep learning algorithms are increasingly being applied to automated liver segmentation, enabling reproducible extraction of quantitative MRI biomarkers and facilitating the generation of regional functional liver maps. Recent deep convolutional neural network models have reported liver segmentation accuracies of approximately 98–99% with Dice similarity coefficients greater than 95%, supporting their integration into quantitative liver imaging workflows [41,42]. More specifically, AI could support quantitative functional liver imaging at different stages of the workflow. Automated liver and segmental segmentation may enable spatially resolved calculation of parameters such as RLE, HUI, CEI, and T1-derived metrics, facilitating the construction of functional maps and reducing the burden of manual ROI placement. AI-based approaches could also integrate regional functional information with liver volumetry, laboratory parameters, and clinical variables, which may be particularly relevant for preoperative assessment by combining the quantity and function of the future liver remnant. In longitudinal imaging, automated co-registration and quantitative comparison of serial examinations could facilitate assessment of regional functional changes after surgery or systemic and locoregional treatments. Finally, multimodal models could combine hepatobiliary MRI-derived functional parameters with morphological imaging and other quantitative biomarkers, although the incremental clinical value of such approaches remains to be demonstrated. However, clinical translation remains limited by dataset bias, heterogeneity in image acquisition and feature extraction, lack of independent external validation, reproducibility concerns, and limited model interpretability. These challenges may be particularly relevant for quantitative hepatobiliary MRI, where differences in scanner platform, acquisition timing, contrast administration, and post-processing may propagate into AI-derived biomarkers. Multicenter validation using harmonized imaging protocols and transparent, reproducible analysis pipelines will therefore be essential before AI-derived functional biomarkers can be incorporated into routine clinical assessment.

Another promising application concerns longitudinal monitoring of liver function during systemic therapy. In oncology patients receiving chemotherapy, MRI-derived biomarkers may detect early hepatotoxicity before conventional laboratory abnormalities become evident [43]. This may be particularly relevant in patients receiving oxaliplatin-based chemotherapy, as cumulative oxaliplatin exposure has been associated with sinusoidal injury, splenic enlargement, and the development of portal hypertension [44]. The principal quantitative MRI-derived biomarkers currently used for liver function assessment, together with their physiological significance, clinical applications, strengths, and limitations, are summarized in Table 1.

## 4. Gadolinium Excretion and Functional Biliary Imaging

The biliary excretion of hepatobiliary contrast agents provides a unique opportunity to assess bile flow and biliary function. Following hepatocyte uptake, gadolinium is secreted into bile canaliculi and transported through the biliary tree, eventually reaching the gallbladder and intestine [19].

**Figure 4:**
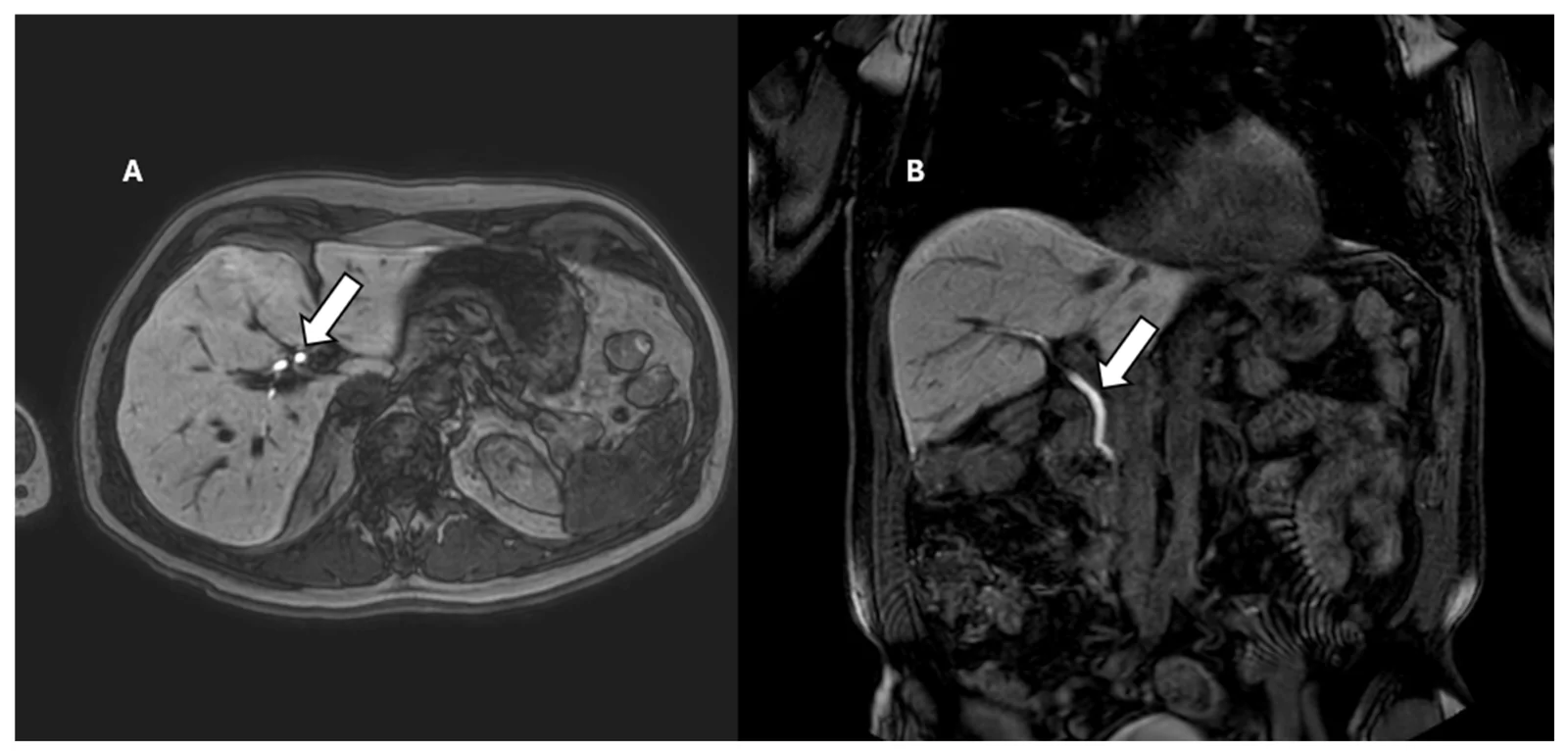
Functional hepatobiliary contrast excretion in a normal liver. (**A**) Axial and (**B**) coronal hepatobiliary phase MRI images obtained after Gd-EOB-DTPA administration demonstrate normal hepatocyte uptake and physiological excretion of contrast material into the intrahepatic and extrahepatic biliary tree (arrows).

This process can be visualized using contrast-enhanced MRI, enabling functional cholangiography. Unlike conventional MR cholangiopancreatography (MRCP), which relies on T2-weighted imaging of static fluid, contrast-enhanced techniques provide dynamic information on bile flow and excretion [45], as shown in Figure 4.

The timing and pattern of biliary enhancement are influenced by several factors, including hepatocyte function, bile production, ductal patency, and pressure gradients within the biliary system. Delayed or absent visualization of the bile ducts may indicate impaired hepatocyte function or biliary obstruction, while early and intense enhancement suggests preserved function [46].

The concentration and temporal appearance of gadolinium within the biliary system represent the integrated result of several processes, including hepatic perfusion, hepatocyte uptake, intracellular transport, canalicular excretion, bile production, ductal patency, and pressure gradients within the biliary tract. Accordingly, biliary gadolinium concentration should not be interpreted as a direct or isolated measure of global liver function. Reduced or delayed biliary enhancement may result from impaired hepatocyte uptake or transporter activity but may also occur in cholestasis, impaired bile production, increased intraductal pressure, or mechanical obstruction. Hepatocellular function, hepatobiliary transport, and biliary drainage should therefore be considered related but biologically distinct components of functional hepatobiliary imaging [46].

Functional biliary imaging has several important clinical applications. In the evaluation of biliary obstruction, it can differentiate between partial and complete obstruction, providing information that may not be apparent on conventional imaging [47]. In postoperative patients, it is highly sensitive for detecting bile leaks, as extravasation of contrast into the peritoneal cavity can be directly visualized [48].

Recent research has increasingly focused on quantitative assessment of hepatobiliary contrast uptake and biliary excretion using dynamic MRI and pharmacokinetic modeling. These approaches enable estimation of quantitative parameters describing hepatocyte uptake, intracellular transport, and biliary excretion kinetics, while accounting for the dual hepatic blood supply and contrast arrival times. Experimental pharmacokinetic models have further demonstrated the feasibility of quantifying hepatobiliary contrast transport and biliary excretion. At the molecular level, OATP1B1/3 mediate hepatocellular uptake of gadoxetic acid, whereas MRP2 mediates canalicular excretion into bile. In contrast, MRP3, located at the basolateral membrane, contributes to efflux from hepatocytes back into the sinusoidal circulation and may therefore influence hepatocellular retention and washout. Collectively, these quantitative approaches have shown promise for the functional assessment of cholestatic liver diseases and drug-induced liver injury [49,50,51,52,53,54].

**Figure 5:**
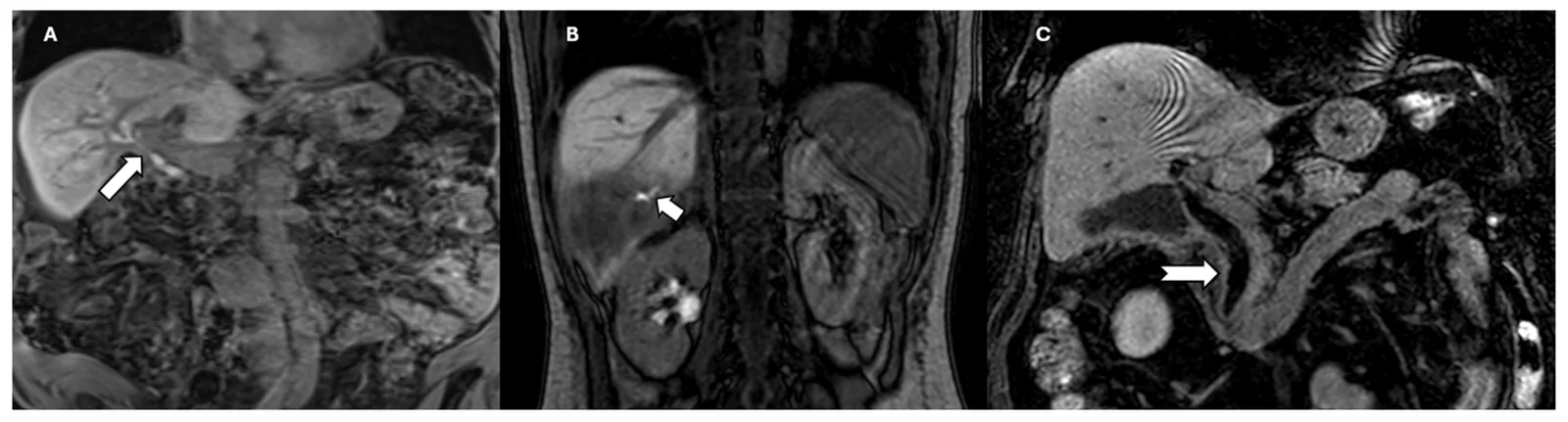
Representative clinical applications of functional hepatobiliary MRI. (**A**) Coronal hepatobiliary phase MRI image showing biliary obstruction at the hepatic hilum (long arrow), with distal contrast excretion. (**B**) Coronal hepatobiliary phase MRI image demonstrating a post-traumatic bile leak (short arrow), with hepatobiliary contrast extravasation into a perihepatic fluid collection. (**C**) Coronal hepatobiliary phase MRI image showing markedly impaired hepatobiliary contrast excretion associated with dilatation of the common bile duct (notched arrow), consistent with biliary outflow obstruction.

Representative examples of functional biliary imaging, including biliary obstruction, bile leak and impaired excretion, are included in Figure 5.

Contrast-enhanced MR cholangiography may provide additional diagnostic information in liver transplant recipients. The combination of conventional T2-weighted MRCP with hepatobiliary contrast-enhanced T1-weighted imaging significantly improves diagnostic confidence for biliary complications, particularly in the detection of anastomotic and non-anastomotic strictures and biliary leaks, compared with conventional MRCP alone. Furthermore, the visualization of hepatobiliary contrast excretion provides complementary functional information on biliary drainage that may aid in the assessment of graft biliary complications [55]. This functional assessment improves diagnostic confidence in the evaluation of biliary strictures, bile leaks, biliary-enteric anastomoses, and other biliary tract abnormalities, complementing conventional MRCP [56].

## 5. MRI in Chronic Liver Disease

Chronic liver disease is characterized by progressive fibrosis, architectural distortion, portal hypertension, and ultimately cirrhosis. These changes profoundly affect hepatocyte function, sinusoidal perfusion, and transporter expression, all of which influence hepatobiliary contrast uptake and excretion [57].

Multiple studies have demonstrated that hepatobiliary phase enhancement progressively decreases with increasing fibrosis severity, reflecting both the loss of functional hepatocytes and impaired hepatocellular transporter activity. For example, the liver-to-spleen signal intensity ratio showed a strong inverse correlation with fibrosis stage (ρ = −0.641) and accurately identified advanced fibrosis (≥F3) with 76.9% sensitivity and 83.3% specificity. Moreover, quantitative enhancement has been shown to be influenced not only by fibrosis but also by hepatic inflammatory activity, indicating that hepatobiliary contrast uptake reflects the combined effects of structural and inflammatory liver injury [23,58,59]. More recently, quantitative T1 mapping has emerged as a robust biomarker of liver function, with T1post and the hepatocyte uptake rate showing strong correlations with ALBI grade (ρ = 0.762 and −0.759, respectively) and excellent performance in discriminating different stages of liver function impairment [60,61].

MRI-derived liver function assessment may therefore complement or potentially reduce the need for invasive liver biopsy. Unlike biopsy, which is prone to sampling error, MRI evaluates the entire liver and may better reflect the heterogeneous distribution of fibrosis. In a prospective validation study, Gd-EOB-DTPA-enhanced MRI accurately discriminated different Ishak fibrosis stages, with relative enhancement-based thresholds achieving sensitivities and positive predictive values of at least 86%, supporting its use as a reliable non-invasive biomarker of liver fibrosis and cirrhosis [62].

Portal hypertension also influences hepatobiliary enhancement. Reduced portal venous inflow and sinusoidal capillarization impair contrast delivery and hepatocyte uptake [63]. In advanced cirrhosis, delayed biliary excretion and reduced hepatobiliary phase enhancement are common findings. Accordingly, MRI-derived functional biomarkers have shown prognostic value, correlating with the severity of liver dysfunction and predicting hepatic decompensation and postoperative outcomes [64].

## 6. MRI in Oncology

In oncology, hepatobiliary MRI has become the reference imaging modality for the detection and characterization of focal liver lesions. Gadoxetic acid-enhanced MRI provides excellent diagnostic performance, with reported areas under the receiver operating characteristic curve (AUROC) of 0.97–0.99 for small hepatocellular carcinoma and approximately 0.98 for colorectal liver metastases [65]. In hepatocellular carcinoma, reduced or absent uptake of hepatobiliary contrast agents during the hepatobiliary phase is a hallmark imaging feature, reflecting loss of hepatocellular function together with downregulation of the organic anion transporting polypeptides OATP1B1 and, predominantly, OATP1B3, which mediate gadoxetate uptake into hepatocytes [66,67]. Within the Liver Imaging Reporting and Data System (LI-RADS), hepatobiliary phase hypointensity is currently considered an ancillary feature favoring malignancy, but it is not a major diagnostic criterion [68,69]. Conversely, several Asian guidelines including the Korean Liver Cancer Association-National Cancer Center (KLCA-NCC) Practice Guidelines, the Japan Society of Hepatology (JSH) Clinical Practice Guidelines, and the Asian Pacific Association for the Study of the Liver (APASL) Clinical Practice Guidelines [70,71,72] assign greater diagnostic weight to hepatobiliary phase hypointensity, particularly for the diagnosis of small hepatocellular carcinomas on gadoxetic acid-enhanced MRI [73,74]. These differences largely reflect the widespread adoption of hepatobiliary contrast-enhanced MRI in Asian countries and a greater emphasis on maximizing sensitivity for early hepatocellular carcinoma detection [75].

Interestingly, a subset of hepatocellular carcinomas demonstrates paradoxical hepatobiliary phase hyperintensity due to preserved or upregulated OATP1B3 expression. These tumors represent a distinct molecular subtype, frequently associated with Wnt/β-catenin pathway activation, well-differentiated histology, and biological characteristics that differ from those of conventional hepatobiliary phase-hypointense hepatocellular carcinomas [76].

For liver metastases, hepatobiliary MRI significantly improves sensitivity, particularly for small lesions. Studies have shown that contrast-enhanced MRI with hepatobiliary phase imaging outperforms CT and non-contrast MRI in detecting liver metastases, with diagnostic accuracies approaching 97–98% in some series [77]. More recently, these advantages have been extended to 18F-fluorodeoxyglucose (FDG) positron emission tomography (PET)/MRI. Although the combination of metabolic PET information with multiparametric MRI already provides excellent diagnostic performance for liver metastases, the addition of hepatobiliary phase imaging further improves sensitivity and specificity, particularly for small or equivocal lesions, resulting in more accurate staging and increased diagnostic confidence [78].

Additional clinical applications include preoperative liver function assessment prior to hepatectomy and monitoring after portal vein embolization [79]. Functional MRI may also be relevant in assessing treatment response after transarterial chemoembolization, radioembolization, or ablative therapies [80]. Residual viable tumors often remain hypointense during the hepatobiliary phase, whereas treated necrotic tissue may demonstrate altered enhancement characteristics [80].

Another emerging application concerns immunotherapy response assessment. Changes in hepatocyte function and regional enhancement patterns may provide additional biomarkers beyond size criteria, potentially improving evaluation of therapeutic efficacy [81].

## 7. Limitations of MRI-Based Functional Imaging

Despite its advantages, MRI-based functional imaging has several limitations. Acquisition times are relatively long, and image quality may be affected by motion artifacts, respiratory motion, or poor breath-holding [82,83]. The availability of MRI is limited in some settings, and contraindications such as implanted devices or severe claustrophobia may preclude its use [84,85].

Concerns regarding gadolinium retention have emerged over the past decade following evidence of gadolinium deposition in the brain, bone, skin, and other tissues, even in patients with normal renal function and after administration of both linear and, to a lesser extent, macrocyclic gadolinium-based contrast agents. Although a causal relationship between tissue retention and long-term clinical symptoms remains unproven, these findings have prompted regulatory actions and renewed interest in optimizing gadolinium-based contrast agent selection, minimizing unnecessary repeated administrations, and developing safer contrast agents and non-gadolinium alternatives [86].

An important limitation to clinical translation is the lack of complete standardization across institutions. Quantitative measurements may be affected by scanner vendor, magnetic field strength, pulse-sequence parameters, contrast dose and injection protocol, hepatobiliary-phase timing, ROI definition, normalization strategy, and post-processing algorithms. Consequently, thresholds derived from individual cohorts may not necessarily be transferable across platforms or institutions. Multicenter validation using harmonized acquisition and analysis protocols is therefore required before universal clinical cutoffs can be proposed. Future efforts should prioritize standardized acquisition timing, reproducible ROI or automated segmentation strategies, vendor-independent quantitative approaches, quality-control procedures, and transparent reporting of post-processing pipelines. Such harmonization will be essential for converting promising imaging biomarkers into reproducible clinical decision-support tools [52]. Signal intensity-based methods may also be influenced by sequence parameters and technical factors [10]. Another limitation concerns patients with severe hepatic dysfunction. In advanced cirrhosis, reduced hepatocyte uptake may significantly impair image quality during the hepatobiliary phase, limiting diagnostic performance [10].

These limitations motivate continued investigation of complementary quantitative imaging approaches, while any alternative technique would require independent validation of technical performance and clinical utility.

Although correlations with Child–Pugh, MELD, ALBI, or ICG clearance support the biological validity of MRI-derived functional biomarkers, correlation alone should not be interpreted as evidence of clinical utility. For clinical adoption, imaging biomarkers should demonstrate incremental value beyond established clinical, laboratory, functional, or volumetric approaches and, ideally, show that their use changes management or improves patient outcomes. Prospective studies comparing imaging-guided and conventional management strategies are therefore required.

## 8. PCCT and K-Edge Imaging

PCCT represents a major advancement in CT technology, offering improved spatial resolution, reduced noise, and spectral imaging capabilities. By measuring the energy of individual photons, photon-counting detectors enable the differentiation of materials based on their energy-dependent attenuation properties [87,88].

In conventional CT systems, energy-integrating detectors measure the total deposited energy, without discriminating individual photon energies. In contrast, photon-counting detectors register each incoming photon and categorize it according to energy thresholds. This allows generation of spectral datasets and material decomposition maps [87,89].

K-edge imaging exploits the abrupt increase in attenuation that occurs when photon energy exceeds the binding energy of inner-shell electrons [90]. For gadolinium, this K-edge is located at approximately 50.2 kiloelectron volts (keV). With appropriate detector architecture, energy thresholds, material-decomposition algorithms, and sufficient gadolinium concentration, photon-counting systems can experimentally discriminate gadolinium on the basis of its spectral properties [91].

This capability has been demonstrated in several preclinical studies, including phantom and animal models, in which photon-counting CT accurately quantified gadolinium concentration with a linear relationship between gadolinium concentration and signal intensity [92,93]. Importantly, demonstration of gadolinium attenuation, K-edge discrimination, quantitative material decomposition, and functional hepatobiliary imaging should not be considered equivalent. Successful gadolinium quantification in phantoms or experimental systems does not establish that clinically achievable hepatic or biliary concentrations can be detected in humans. In this context, Baubeta et al. [16] reported no detectable gadolinium K-edge on a first-generation clinical photon-counting CT system under the investigated conditions, despite testing concentrations considered relevant to clinical use. This negative result highlights the substantial gap between experimental material discrimination and clinically feasible hepatobiliary K-edge imaging. Experimental and preclinical studies have demonstrated gadolinium K-edge angiography under selected conditions, primarily in vascular rather than hepatobiliary applications [94,95].

**Figure 6:**
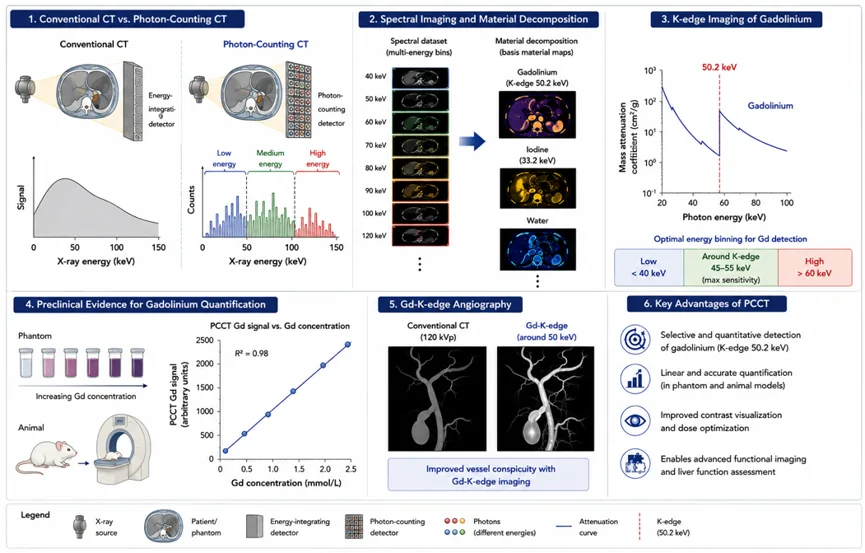
Principles of photon-counting CT and gadolinium K-edge imaging. Illustration of photon-counting detector technology, energy discrimination, K-edge imaging of gadolinium, material decomposition, and generation of gadolinium-specific maps for potential quantitative assessment of hepatobiliary contrast distribution.

The physical principles underlying PCCT and K-edge imaging, including the selective detection of gadolinium, are illustrated in Figure 6.

PCCT additionally offers several advantages beyond K-edge imaging. These include improved contrast-to-noise ratio, reduction in electronic noise, decreased blooming artifacts, and improved spatial resolution [88]. Material-decomposition algorithms have enabled experimental separation of iodine and gadolinium in phantom and preclinical studies. Whether such dual-contrast approaches can combine vascular and hepatobiliary information at clinically acceptable doses remains to be established [96].

## 9. Gadolinium Imaging with PCCT

Phantom studies have shown a linear relationship between gadolinium concentration and CT signal, enabling quantitative imaging. Animal studies have further demonstrated the ability to visualize gadolinium-enhanced structures using K-edge imaging [94,95,96,97].

These experimental findings provide a technical rationale for investigating whether selected aspects of hepatobiliary gadolinium distribution might eventually become measurable with PCCT; however, hepatocyte-specific uptake and biliary excretion have not yet been demonstrated as quantitative functional endpoints in human hepatobiliary PCCT [98].

The concentration of gadolinium used in MRI is substantially lower than iodine concentrations typically employed in CT imaging [99]. This concentration mismatch is quantitatively relevant. In a phantom study specifically evaluating gadoxetate disodium, concentrations of 0.250–2.5 μmol/mL were investigated, corresponding to modeled administered doses of approximately 25–200 μmol/kg. At 40 keV, the highest concentration yielded an attenuation of 45.2 HU, whereas 0.25 μmol/mL produced 13.0 HU [14]. Thus, substantial enhancement was mainly observed at concentrations exceeding those expected after standard clinical administration. More recently, dedicated multi-threshold PCCT experiments demonstrated quantitative gadolinium material decomposition at concentrations of 1–10 mg/mL and radiation doses of 1–8 mGy, with performance improving with increasing concentration and dose [99]. These findings establish technical detectability under selected experimental conditions but do not establish that hepatic or biliary gadolinium concentrations after standard gadoxetate administration are sufficient for reliable human K-edge imaging.

Indeed, reliable quantitative mapping of hepatobiliary gadolinium after standard clinical administration has not yet been demonstrated [95,100]. Current levels of evidence for gadolinium detection, K-edge imaging, and potential hepatobiliary applications of photon-counting CT are summarized in Table 2.

Another important consideration concerns radiation dose. Although PCCT may improve dose efficiency compared with conventional CT, repeated functional imaging studies may still expose patients to ionizing radiation. Careful optimization of acquisition parameters will therefore be essential [101]. The complementary characteristics of hepatobiliary MRI and PCCT for functional liver imaging are summarized in Table 3.

## 10. Translational Requirements for Potential CT-Based Hepatobiliary Functional Imaging

Functional CT assessment of the liver is not entirely novel, as previous studies have already explored quantitative CT biomarkers for the evaluation of hepatic fibrosis and extracellular volume using contrast-enhanced CT [102,103,104,105]. The spectral capabilities of PCCT provide a technical platform for investigating gadolinium-specific material decomposition. Whether this capability can be translated into measurement of hepatocyte uptake or biliary excretion at clinically achievable concentrations remains unproven. Such an approach could leverage several intrinsic advantages of PCCT, including rapid image acquisition, reduced susceptibility to motion artifacts, and improved dose efficiency compared with conventional CT. However, the extent to which these advantages would translate to gadolinium-based functional hepatobiliary imaging remains to be established, particularly if delayed acquisitions are required.

Other challenges must nevertheless be addressed. The concentration of gadolinium in bile is relatively low, requiring high detector sensitivity and optimized imaging protocols [19]. Radiation exposure is another consideration, particularly in patients requiring repeated imaging [101]. The gadolinium concentrations encountered in hepatic parenchyma and bile following clinically approved doses may approach the limits of reliable material discrimination, and the minimum detectable concentration under clinically acceptable radiation-dose conditions remains to be established. Translation will therefore require optimization of detector sensitivity, energy thresholds, contrast dose and timing, as well as prospective human validation. Repeated or delayed CT acquisitions would additionally need to demonstrate an acceptable radiation burden compared with the radiation-free functional information provided by MRI. Finally, protocols involving alternative gadolinium doses or indications would require appropriate safety and regulatory evaluation. Accordingly, gadolinium K-edge PCCT should currently be regarded as an experimental proof-of-concept rather than a clinically validated alternative to hepatobiliary MRI. A further translational issue is whether currently approved hepatobiliary gadolinium agents are intrinsically suitable for PCCT. Gd-EOB-DTPA was developed and dosed for MR relaxivity and hepatocyte-specific pharmacokinetics rather than for X-ray K-edge discrimination. Increasing the administered dose solely to achieve CT-detectable concentrations would require independent evaluation of pharmacokinetics, toxicity, renal safety, tissue retention, and cumulative gadolinium exposure. Modified formulations or dedicated gadolinium-containing/high-Z agents optimized for photon-counting CT may therefore ultimately be required. Such agents would require dedicated preclinical toxicology, dose-finding studies, and regulatory approval and would represent a new contrast-enhanced CT application rather than a simple translation of the existing hepatobiliary MRI protocol.

Future research should first establish the minimum detectable hepatobiliary gadolinium concentration under clinically acceptable radiation conditions, determine whether standard gadoxetate dosing is sufficient, and assess whether dedicated contrast agents or detector/reconstruction strategies are required.

Possible scenarios include patients unable to undergo MRI, combined vascular and delayed hepatobiliary assessment in selected preoperative settings, regional functional mapping when CT is already clinically indicated, and selected biliary complications. For each scenario, future studies would need to demonstrate not only technical feasibility but also incremental diagnostic or prognostic value over hepatobiliary MRI, conventional CT, laboratory-based scores, ICG clearance, elastography, and volumetric assessment. Any potential benefit would additionally need to justify the radiation exposure, contrast-agent dose, and acquisition complexity required by the PCCT protocol.

## 11. Conclusions

MRI-derived evaluation of liver function using hepatobiliary contrast agents represents a significant advancement in medical diagnostics, enabling the non-invasive assessment of hepatocyte function and biliary excretion. The temporal distribution of hepatobiliary gadolinium provides complementary information on hepatocyte uptake, hepatobiliary transport, and biliary drainage, although these processes should not be regarded as interchangeable measures of global liver function.

Quantitative MRI approaches, including RLE, T1 mapping, pharmacokinetic modeling, and functional mapping, have expanded the range of functional information obtainable from hepatobiliary imaging and are increasingly being investigated in chronic liver disease, oncologic imaging, surgical planning, and treatment monitoring.

PCCT and gadolinium K-edge imaging represent an experimental extension of these concepts rather than an established functional liver-imaging technique. Although gadolinium attenuation, material decomposition, and K-edge imaging have been demonstrated under selected phantom, preclinical, and limited non-hepatobiliary human conditions, quantitative hepatocyte uptake and biliary excretion imaging with PCCT have not been validated in humans. Major unresolved issues include the low gadolinium concentrations achieved with standard hepatobiliary contrast dosing, detector and reconstruction requirements, radiation exposure, contrast-agent suitability, safety, and regulatory considerations. A clinical role can therefore only be considered if future studies demonstrate both technical feasibility and meaningful incremental value over established MRI and non-imaging functional assessments.

The future of hepatobiliary imaging is likely to be increasingly quantitative and personalized, but the clinical translation of emerging imaging biomarkers and technologies will require rigorous prospective validation, standardization, and demonstration of incremental value over established diagnostic strategies.

## Figures and Tables

**Table 1:** Quantitative MRI-derived biomarkers currently used for liver function assessment. Summary of the principal quantitative biomarkers derived from hepatobiliary MRI, including their calculation, physiological significance, main clinical applications, strengths, and limitations.

Biomarker	Reflects	How It Is Calculated	Current Evidence	Main Limitations	Main Strengths	Main Clinical Applications
Relative Liver Enhancement (RLE)	Hepatocyte uptake	% signal increase from precontrast to HBP	Established correlations with ALBI, MELD and Child–Pugh	Sequence-, scanner-, timing-, and signal-normalization-dependent	Simple, reproducible	Cirrhosis, postoperative liver failure prediction
Liver-to-Spleen Ratio	Relative hepatic enhancement	Liver SI/spleen SI	Associations with fibrosis, Child–Pugh and MELD	Affected by splenic abnormalities, ROI placement, and acquisition parameters	Easy to calculate	Chronic liver disease
Hepatic Uptake Index (HUI)	Hepatocyte extraction	Liver enhancement normalized to blood pool	Higher discrimination than volumetric assessment reported in selected cohorts; incremental clinical utility requires further validation	Slightly more complex	Promising prognostic performance	Surgical planning, postoperative liver failure
Contrast Enhancement Index (CEI)	Hepatic enhancement	Enhancement normalized to internal reference	Quantitative assessment of hepatic enhancement and liver dysfunction, particularly in chronic liver disease	Reference-tissue selection and calculation methodology are not standardized	Simple quantitative metric	Chronic liver disease
T1 mapping	Hepatocyte uptake	ΔT1, T1post, rrT1, Khep	Strong correlation with liver-function measures	Requires dedicated sequences; affected by field strength, sequence implementation, and vendor-specific methodology	Highly quantitative	Liver function assessment
Pharmacokinetic modeling	Uptake + excretion kinetics	K1, K2, uptake rate, excretion rate	Predominantly research setting	Complex acquisition and modeling; limited cross-platform and multicenter standardization	Physiological modeling	Research, advanced functional imaging

**Table 2:** Current evidence for gadolinium detection, K-edge imaging, and potential hepatobiliary applications of photon-counting CT. PCCT, photon-counting computed tomography; PCD-CT, photon-counting detector computed tomography; GBCA, gadolinium-based contrast agent; Gd-EOB-DTPA, gadoxetate disodium; Gd, gadolinium; CNR, contrast-to-noise ratio. Demonstration of gadolinium attenuation, K-edge discrimination, or material decomposition should not be considered equivalent to demonstration of hepatocyte uptake, biliary excretion, or regional liver function.

Evidence Level	Experimental/Clinical Setting	What Has Been Demonstrated	What Has Not Been Demonstrated	Main Translational Limitations
Human clinical hepatobiliary MRI	Clinical practice	Hepatocyte-specific uptake, biliary excretion, regional functional assessment, and quantitative MRI-derived biomarkers	—	MRI-specific limitations, including acquisition time, motion sensitivity, and incomplete biomarker standardization
Human PCCT–gadolinium imaging	Limited technical/clinical experience	Gadolinium-related attenuation and, under selected technical conditions, human K-edge imaging outside the hepatobiliary setting	Hepatocyte-specific uptake, quantitative biliary excretion, or regional liver function using hepatobiliary gadolinium agents	Limited human evidence; dependence on detector architecture, energy thresholds, gadolinium concentration, and reconstruction
Preclinical animal PCCT	Animal experiments	Gadolinium visualization, K-edge imaging, vascular applications, and experimental dual-contrast imaging	Validation of human hepatobiliary functional imaging	Experimental dosing, animal physiology, and uncertain translation to clinically achievable human concentrations
Phantom PCCT	Phantom studies	Gadolinium detectability, concentration-dependent attenuation, material decomposition, K-edge discrimination under selected acquisition conditions	Biological uptake, biliary transport, or clinical functional assessment	Optimized experimental conditions; often higher gadolinium concentrations and dedicated acquisition/reconstruction protocols
Simulation/theoretical studies	Computational models	Theoretical feasibility of spectral discrimination and optimization of detector thresholds/material decomposition	Clinical feasibility and biological validation	Results depend on assumed detector performance, noise, dose, and material concentrations
Human Gd-EOB-DTPA hepatocyte uptake with K-edge PCCT	Not yet established	—	Direct quantitative demonstration of hepatocyte-specific uptake	Unknown sensitivity at clinically achievable hepatic gadolinium concentrations
Human quantitative biliary gadolinium excretion with PCCT	Not yet established	—	Quantitative measurement of biliary gadolinium excretion	Unknown biliary concentration, detection threshold, CNR, radiation dose, and timing requirements
PCCT-derived regional liver function	Not yet established	—	Validated regional functional liver mapping based on hepatobiliary gadolinium	Requires technical feasibility, biological validation, reproducibility, and demonstration of incremental clinical value

**Table 3:** Comparison of established hepatobiliary MRI capabilities with current evidence and potential applications of photon-counting CT.

Feature	Hepatobiliary MRI	PCCT—Current Evidence	Interpretation/Limitations
Hepatocyte-specific uptake	Established in humans	Not demonstrated as a functional PCCT endpoint in humans	Potential application remains hypothetical
Biliary excretion imaging	Established in humans	Not demonstrated with human Gd-based PCCT	Potential application remains hypothetical
Regional liver function assessment	Clinically investigated using multiple quantitative MRI biomarkers	Not clinically validated	Requires demonstration of both technical feasibility and biological validity
Quantitative functional biomarkers	RLE, HUI, CEI, T1-based parameters, and pharmacokinetic modeling	No validated Gd-based functional biomarker	MRI biomarkers have substantially greater clinical evidence and validation
Gadolinium detection	Assessed indirectly through its effect on MR signal	Gd-related attenuation demonstrated experimentally	Detection does not imply K-edge discrimination or functional imaging
Gadolinium K-edge discrimination	Not applicable	Demonstrated under selected experimental conditions, with variable performance across systems	Strongly dependent on Gd concentration, detector architecture, energy thresholds, dose, and reconstruction
Quantitative Gd material decomposition	Not directly concentration-based in routine MRI	Demonstrated in selected phantom/preclinical settings	Not validated at human hepatobiliary concentrations after standard Gd-EOB-DTPA dosing
Dual-contrast iodine/Gd imaging	Not routinely applicable	Experimentally demonstrated	Clinical hepatobiliary utility remains unproven
Acquisition time	Multiparametric examination with delayed hepatobiliary phase	Individual CT acquisitions are rapid	Functional PCCT may require delayed/repeated acquisitions; overall workflow advantage is unproven
Motion sensitivity	Relevant, particularly for dynamic and quantitative acquisitions	Lower for individual CT acquisitions	Potential advantage may be reduced by multiphase or delayed functional protocols
Radiation exposure	None	Present; PCCT may provide improved dose efficiency compared with conventional CT	Particularly relevant if repeated or delayed acquisitions are required
Contrast-agent suitability	Hepatobiliary GBCAs clinically approved for MRI	Current hepatobiliary GBCAs are not optimized or approved specifically for CT K-edge imaging	Higher doses or dedicated agents would require pharmacological, safety, and regulatory evaluation
Clinical availability	Established	Clinical PCCT available in selected centers	Gd-based functional hepatobiliary PCCT is not clinically available
Current clinical role	Established for morphological and functional hepatobiliary assessment	No established functional hepatobiliary indication	Currently experimental/research only

## Data Availability

No new data were created or analyzed in this study. Data sharing is not applicable to this article.

## Abbreviations

ALBI	Albumin–Bilirubin
APASL	Asian Pacific Association for the Study of the Liver
ATP	Adenosine Triphosphate
AUC	Area Under the Curve
AUROC	Area Under the Receiver Operating Characteristic Curve
CEI	Contrast Enhancement Index
CT	Computed Tomography
DCNN	Deep Convolutional Neural Network
DCE-MRI	Dynamic Contrast-Enhanced Magnetic Resonance Imaging
FDG	Fluorodeoxyglucose
GBCA	Gadolinium-Based Contrast Agent
Gd-BOPTA	Gadobenate Dimeglumine
Gd-EOB-DTPA	Gadoxetate Disodium (Gadoxetic Acid)
HBP	Hepatobiliary Phase
HCC	Hepatocellular Carcinoma
HUI	Hepatic Uptake Index
ICC	Intraclass Correlation Coefficient
ICG	Indocyanine Green
ICG-R15	Indocyanine Green Retention Rate at 15 Minutes
JSH	Japan Society of Hepatology
Khep	Hepatic Uptake Rate Constant
KLCA-NCC	Korean Liver Cancer Association–National Cancer Center
LI-RADS	Liver Imaging Reporting and Data System
LSR	Liver-to-Spleen Signal Intensity Ratio
MELD	Model for End-Stage Liver Disease
MRP2	Multidrug Resistance-Associated Protein 2
MRP3	Multidrug Resistance-Associated Protein 3
MRCP	Magnetic Resonance Cholangiopancreatography
MRI	Magnetic Resonance Imaging
OATP	Organic Anion Transporting Polypeptide
PCCT	Photon-Counting Computed Tomography
PET	Positron Emission Tomography
RLE	Relative Liver Enhancement
ROI	Region of Interest
T1post	Post-contrast T1 Relaxation Time
T1pre	Pre-contrast T1 Relaxation Time
ΔT1	Change in T1 Relaxation Time
rrT1	T1 Reduction Rate
